# Novel role of ASC as a regulator of metastatic phenotype

**DOI:** 10.1002/cam4.800

**Published:** 2016-06-28

**Authors:** Nagisa Okada, Chifumi Fujii, Tomio Matsumura, Masato Kitazawa, Ryuhei Okuyama, Shun'ichiro Taniguchi, Shigeaki Hida

**Affiliations:** ^1^ Department of Molecular OncologyInstitute of Pathogenesis and Disease PreventionGraduate School of MedicineShinshu UniversityAsahi 3‐1‐1Matsumoto390‐8621Japan; ^2^ Department of DermatologySchool of MedicineShinshu UniversityAsahi 3‐1‐1Matsumoto390‐8621Japan; ^3^ Department of Advanced Medicine for Health PromotionInstitute for Biomedical SciencesInterdisciplinary Cluster for Cutting Edge ResearchShinshu UniversityAsahi 3‐1‐1Matsumoto390‐8621Japan; ^4^ Department of SurgerySchool of MedicineShinshu UniversityAsahi 3‐1‐1Matsumoto390‐8621Japan; ^5^ Department of Comprehensive Cancer TherapySchool of MedicineShinshu UniversityAsahi 3‐1‐1Matsumoto390‐8621Japan; ^6^ Department of Molecular and Cellular Health ScienceGraduate School of Pharmaceutical SciencesNagoya City University3‐1 Tanabe‐doriMizuho‐kuNagoya467‐8603Japan

**Keywords:** ASC, invadopodia, metastasis, motility, Src signaling pathway

## Abstract

Disorders of cytoskeletal remodeling and signal transduction are frequently involved in cancer progression. In particular, apoptosis‐associated speck‐like protein containing a caspase‐recruitment domain (ASC) has been reported a proapoptotic molecule that is epigenetically silenced in several human cancers. ASC is a well‐characterized adaptor protein involved in the formation of multiprotein oligomers, called inflammasomes, and plays a crucial role in the activation and secretion of interleukin‐1*β* and interleukin‐18 in innate immune cells. However, the function of ASC in the regulation of tumor progression remains elusive. The present investigation examined the involvement of ASC in cancer progression and the acquisition of metastatic ability. To determine the effect of ASC depletion in in vitro and in vivo model systems, ASC was stably knocked down in B16 murine melanoma cell lines using retroviral transduction of shRNA. ASC suppression increased the motility of B16BL6 cells in scratch assays and augmented invasiveness as assessed by a Matrigel‐coated transwell system. Invadopodia formation and Src phosphorylation level were markedly enhanced in ASC‐knockdown cells as well. Since caspase‐8 has been reported to enhance cellular migration by Tyr380 phosphorylation via Src, we examined Tyr380 phosphorylation of caspase‐8 in ASC‐knockdown cells and found it to be elevated in ASC‐knockdown cells but attenuated by z‐VAD‐fmk or z‐IETD‐fmk. Moreover, ASC ablation increased pulmonary metastasis in mice after intravenous injection of B16BL6 cells. Our cumulative findings indicate that ASC suppresses cancer metastasis and progression via the modulation of cytoskeletal remodeling and the Src‐caspase‐8 signaling pathway.

## Introduction

Apoptosis‐associated speck‐like protein containing a caspase‐recruitment domain (ASC) was originally identified as a component of the Triton X‐100‐insoluble fraction, called “speck”, of a retinoic acid‐treated HL‐60 cell line 1. Nowadays, ASC is well known as a key adaptor protein in the formation of various inflammasomes that plays crucial roles in caspase‐1 activation and the secretion of interleukin (IL)‐1*β* and IL‐18 in innate immune cells 2, 3, 4. On the other hand, ASC has also been identified as a target of methylation‐induced silencing 1 (TMS1) and one of the genes silenced by the overexpression of DNA methyltransferase in breast cancer 5. Referred to as well as PYCARD since it contains a pyrin homologous domain (PYD) and caspase‐recruitment domain (CARD) 3, ASC appears to have numerous identities and thus is widely studied in the fields of inflammatory response, epigenetics, and tumor biology 6.

Accumulating evidence has suggested that the suppression of ASC by methylation results in a poor prognostic tendency in multiple human cancers. We previously reported that ASC expression was reduced in melanoma 7, colorectal cancer 8, lung cancer 9, and oral squamous cell carcinoma (OSCC) 10. In lung cancer, diminished expression of ASC was correlated with the invasive stages of tumor progression, and the *ASC* promoter was significantly hypermethylated in invasive lung adenocarcinoma patients with metastasis to the lymph nodes 9. Furthermore, our recent study indicated that ASC expression was significantly lower in nonborder invasive and diffusion type tissues than in noninvasive type tumors in OSCC 10. Other groups have also identified relationships between silencing of the *ASC* gene by methylation and prognosis in prostate cancer 11, 12, glioblastoma 13, hepatocellular carcinoma 14, cervical cancer 15, and others. Recently, Liu et al. 16 demonstrated that ASC was epigenetically inactivated in 41.1% of renal cell carcinoma (RCC) and suggested a role of tumor suppressor. Wu et al. 17 reported that hypermethylation of the *ASC/TMS1* promoter was significantly associated with greater lymph node metastasis, associated with a poor prognosis in patients with gastric cancer, and should be considered as a key prognostic indicator.

We earlier traced a reduction in ASC expression in human melanoma to *ASC* gene downregulation by aberrant methylation 7. Specifically, ASC expression was reduced in 62.5% (20 of 32) of melanoma tissues and 58.3% (7 of 12) of melanoma cell lines 7. These observations prompted us to examine the relationships between reductions in ASC expression levels and cancer cell malignancy, that is, the acquisition of metastasis. By hypothesizing that a deficiency in ASC expression influenced the metastatic properties of cancer cells, we employed RNA interference to reduce ASC expression and mimic gene silencing by methylation in B16 melanoma cell lines, and thereafter analyzed their phenotypes and molecular events both in vitro and in vivo.

## Materials and Methods

### Antibodies and reagents

Anti‐murine ASC rabbit polyclonal antibodies were prepared as described previously 18. Antibodies against Src, phospho‐Src family kinases (Tyr416), Akt (pan), phospho‐Akt (Ser473), Erk 1/2, phospho‐Erk 1/2 (Tyr202/Tyr204), p38 MAPK, phospho‐p38 MAPK (Tyr180/Tyr182), SAPK/JNK, phospho‐SAPK/JNK (Tyr183/Tyr185), and caspase‐8 were all purchased from Cell Signaling Technologies (Beverly, MA). Antibodies against FAK and phospho‐FAK (Tyr397) were obtained from GenTex (Irvine, CA). Anti‐phospho‐caspase‐8 (pTyr380) and anti‐*β*‐actin antibodies were procured from Sigma (St. Louis, MO). All other reagents and chemicals were purchased from Sigma, Wako (Osaka, Japan), or Nacalai Tesque (Kyoto, Japan) unless otherwise specified.

### Cell culture and proliferation analysis

B16BL6 and B16F10 murine melanoma cell lines 19 were maintained in Dulbecco's modified Eagle's medium with high glucose (DMEM high glucose; Sigma‐Aldrich) supplemented with 10% heat‐inactivated fetal bovine serum (FBS; BioWest, Nuaillé, France) at 37 °C in a humidified 5% CO_2_ atmosphere. The retroviral packaging cells were cultured in DMEM (Sigma‐Aldrich) containing 10% FBS at 37 °C in a 5% CO_2_ humidified atmosphere. Cell number and viability were counted under a light microscope using the trypan blue dye exclusion assay.

### Knockdown of ASC

ASC‐knockdown vectors were constructed using pSINsi‐hU6 short hairpin RNA (shRNA) expression retroviral vectors (Takara Bio, Shiga, Japan) modified with a hygromycin‐B resistance gene instead of a neomycin marker. The target sequence for murine ASC corresponds to coding region 430–448 (5′‐GCTTAGAGACATGGGCTTA‐3′). The negative control sequence was provided from Takara Bio. For infection with retroviral vectors, retroviral packaging cells cultured in 100 mm dishes were incubated with 6 *μ*g of recombinant retroviral vector and 21 *μ*g of Polyethyleneimine “MAX” (Polysciences, Inc., Warrington, UK) for 8 h. Thereafter, the cells were cultured in fresh DMEM with 10% FBS for 48 h, during which time the supernatant containing the retroviral particles was collected twice. The supernatants were concentrated by centrifugation at 6000*g* for 16 h, filtered through a 0.45 *μ*m filter, and used to infect target cells. Twelve‐well culture plates were coated with 50 *μ*g/mL of RetroNectin (Takara Bio) as recommended by the manufacturer. One day later, 2 mL of concentrated virus supernatant were added to each well, and plates were centrifuged at 3370*g* for 4 h at 32°C. The virus supernatant was removed and replaced by B16BL6 or B16F10 cells (2 × 10^4^ cells/well) in DMEM high glucose with 10% FBS, and cells were incubated at 37 °C for 48 h. The infected cells were then subcultured at an appropriate density in fresh DMEM high glucose containing 0.5 mg/mL hygromycin‐B. Hygromycin‐B‐resistant cell pools were readily established within 10 days.

### Quantitative real‐time reverse transcription polymerase chain reaction (qRT‐PCR)

Total RNA was extracted with RNAiso Plus (Takara Bio) followed by phenol/chloroform extraction and then reverse‐transcribed with Prime Script RT Master Mix (Takara Bio) according to the manufacturer's instructions. We performed qRT‐PCR using SYBR premix Ex Taq II (Takara Bio) in a TP850 Thermal Cycler Dice Real Time System Single (Takara Bio). Relative expression to GAPDH was calculated according to the delta delta ct method.

### Western blotting

Cultivated cells were washed twice with cold phosphate‐buffered saline (PBS) and harvested by centrifugation at 1150*g* at 4°C. The pellet was homogenized in cell lysis buffer (20 mmol/L HEPES‐OH, pH 7.9, 300 mmol/L NaCl, 1 mmol/L EDTA, 15% glycerol, 0.5% Nonidet P‐40, 1 mmol/L Na_3_VO_4_, 10 mmol/L NaF, 1 mmol/L phenylmethylsulfonyl fluoride, and 1 unit of complete protease inhibitor cocktail EDTA‐free (Roche, Pleasanton, CA)) and incubated with gentle rocking at 4°C. After homogenates were centrifuged, the supernatants were saved as cell lysates and stored at −80°C until use.

Protein samples were separated by SDS‐PAGE and electrophoretically transferred to polyvinylidene difluoride membranes. The membranes were blocked in 5% nonfat dry milk in TBS‐T buffer (50 mmol/L Tris‐HCl, pH 7.5, 150 mmol/L NaCl, and 0.1% Tween 20) for 1 h at room temperature and thereafter incubated with primary antibodies in 5% bovine serum albumin (BSA) in TBS‐T overnight at 4°C. The membranes were then washed in TBS‐T and incubated for 1 h at room temperature with horseradish peroxidase‐conjugated secondary antibodies (DAKO, Glostrup, Denmark) in TBS‐T with 1% nonfat dry milk. Detection was carried out with the enhanced chemiluminescence detection system Immobilon Western chemiluminescent HRP substrate (Merck Millipore, Berlin, Germany). All data were processed and analyzed using Printgraph (ATTO, Tokyo, Japan) and CS Analyzer software (ATTO) or myECL Imager (Thermo Fisher Scientific, Rockford, IL) and myImage Analysis software (Thermo Fisher Scientific), respectively.

### In vivo tumorigenesis assay

Female C57BL/6j mice of 8–12 weeks of age were implanted subcutaneously with B16BL6 cells into the flank (1 × 10^6^ cells/site), and then tumor size was measured three times per week using calipers. Tumor volume was calculated as 1/2 (*X* ^2^ *Y*), where *X* and *Y* were the diameters (mm) of two orthogonal measurements and *X* was shorter than *Y*. The mice were killed on day 14 and their tumors harvested and weighed. All animal experiments were performed with the approval of the Institutional Animal Care and Use Committee of Shinshu University School of Medicine.

### Experimental lung metastasis model

Female C57BL/6j mice of 8–12 weeks of age were inoculated intravenously with B16BL6 tumor cells (1 × 10^5^ cells/inoculation). The mice were killed on day 14, their lungs harvested, and the number of surface tumor nodules counted under a stereomicroscope.

### Scratch assay

Aliquots of 4 × 10^6^ cells were seeded onto 60 mm dishes. Six hours later, cell layers were wounded in cultivated medium (DMEM with 10% FBS and high glucose) using a sterile 200 *μ*L tip. After washing away the suspended cells, the remaining cells were cultured in DMEM with 0.1% FBS and low glucose. Migration progress was photographed in six regions, immediately (0 h) and 14 h after wounding under an inverted microscope (EVOS; AR BROWN, Tokyo, Japan). The wounded areas were measured by ImageJ software (http://rsb.info.nih.gov/ij/) and the percent of wound closure was calculated for each area.

### Invasion assay

Transwell cell culture inserts (membrane pore size: 8.0 *μ*m; BD Falcon, Bedford, MA) were coated with 50 *μ*g of Matrigel (BD Biosciences, San Jose, CA). Fifty thousand cells in serum‐free DMEM were seeded in the upper chamber of the inserts. The bottom chamber contained DMEM with 10% FBS as a chemoattractant. After incubation for 48 h, the cells in the upper surface of the membranes were removed with a cotton swab. Cells that had invaded to the lower surface of the membranes were fixed with methanol and stained with 0.2% crystal violet. The invading cells were observed under a light microscope and counted in five randomly selected fields for each well.

### In vitro cell adhesion assay

Ninety‐six well plates were precoated with 0.3 mg/mL collagen type I (Nitta Gelatin, Osaka, Japan), 0.3 mg/mL collagen type IV (Nitta Gelatin), 50 *μ*g/mL fibronectin (Sigma), FBS (BioWest), or 20 *μ*g/mL laminin (Sigma) and then blocked with DMEM containing 0.5% BSA for 1 h. Thereafter, 2 × 10^4^ cells were seeded onto the coated plates and incubated for 30 min. Unadhered cells were removed by washing with DMEM containing 0.1% BSA. Adherent cells were fixed with 4% paraformaldehyde (PFA), stained with 0.5% crystal violet, and washed. Finally, crystal violet was solubilized with 2% SDS, and absorbance of 595 nm (A_595_) was determined by a microplate reader.

### F‐actin detection

B16BL6 cells were cultivated on fibronectin‐coated coverslips (15 mm in diameter) for 24 h. The cells were treated with PBS containing 4% PFA, permeabilized with 0.1% Triton X‐100 in PBS, and supplemented with 100 nmol/L rhodamine‐phalloidin (Cytoskeleton, Inc., Denver, CO) in PBS containing 0.1% BSA for 1 h at room temperature. Lastly, the cells were mounted with VECTASHIELD containing 4,6‐diamidino‐2‐phenylindole dilactate (DAPI) (VECTOR Laboratories, Burlingame, CA), and signals were observed under a confocal microscope (LSM 5 EXCITER; Carl Zeiss, Jena, Germany).

### Gelatin invadopodia assay

Invadopodia assay was performed with QCM Gelatin Invadopodia assay kit (Millipore) according to the manufacturer's instructions. Briefly, cells were cultivated on Cy3‐gelatin‐coated Lab‐Tec chamber slides (Nalge NUNC, Roskilde, Denmark) for 5 h and then fixed by 4% PFA in PBS. Afterward, the fixed cells were stained with fluorescein isothiocyanate (FITC)‐phalloidin and DAPI in PBS containing 2% BSA and 0.25% Triton‐X‐100 before mounting with VECTASHIELD (VECTOR Laboratories) and visualization under a confocal microscope. To quantitate the gelatin degradation activity of invadopodia, signals were observed under a fluorescence microscope (AxioObserverZ1; Carl Zeiss), analyzed with ImageJ software, and normalized to the total cell number in each image. Twenty randomly selected fields were imaged and analyzed for each sample. The value of control cells was set as 1, and then relative ASC‐knockdown cell values were calculated accordingly.

### High‐content imaging Invadopodia assay

B16F10 cells (1 × 10^4^ cells) were plated on Cy3‐gelatin‐coated Glass Bottom ViewPlate‐96F (PerkinElmer, Waltham, MA) for 8 h and fixed by 4% PFA in PBS. Afterward, the fixed cells were stained with FITC‐phalloidin and DAPI in PBS containing 2% BSA and 0.25% Triton‐X‐100. Following washing with PBS, the cells were visualized with the Operetta high‐content imaging system (PerkinElmer) at 20× magnification with 12 fields of view per well. Images were analyzed with Harmony software (PerkinElmer).

### Statistical analysis

Statistical analysis of data was performed using the Student's *t*‐test. Results are expressed as the mean ± standard deviation (SD). A *P* value of <0.05 was accepted as statistically significant.

## Results

### ASC‐knockdown did not affect cellular proliferation but enhanced experimental lung metastasis of B16BL6 cells

**Figure 1:**
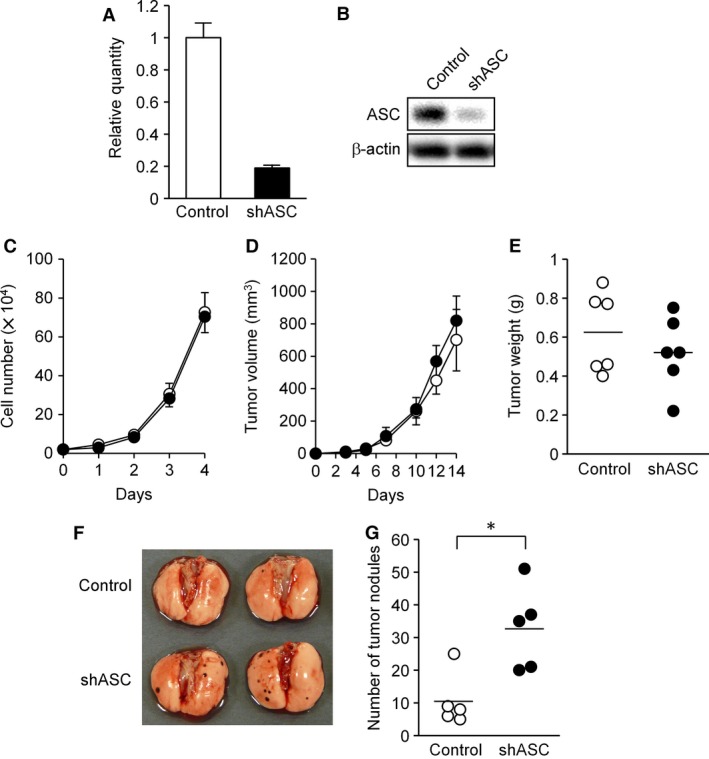
ASC silencing did not affect the cellular proliferation of B16BL6 cells. (A and B) Establishment of ASC‐knockdown B16BL6 cells. A decreased level of ASC was confirmed by qPCR (A) and immunoblot analysis (B). (C) In vitro cellular proliferation analysis. Cells were seeded at 2 × 10^4^ per well in 12‐well plates. Cells from triplicate wells were trypsinized and manually counted for each time point. Trypan blue dye exclusion was more than 98% until day 4 in both cell lines. Results are expressed as the mean (*n* = 3) and error bars indicate SD. (D) Cellular tumorigenesis in the syngeneic mice. C57BL/6j mice were implanted subcutaneously with B16BL6 cells into the flank (1 × 10^6^ cells/site) and tumor size was measured at the indicated time points. Results are expressed as the mean (*n* = 6) and error bars indicate SD. (E) Tumor weights from the mice in experiment D at day 14. Results are expressed as individual points (circles) and bars indicate the mean (*n* = 6). (F and G) Experimental lung metastasis of ASC‐knockdown B16BL6 cells. C57BL/6j mice were inoculated intravenously with B16BL6 cells (5 × 10^4^ cells). The animals were sacrificed on day 14, their lungs harvested, and the number of tumor nodules counted under a stereomicroscope. (F) Representative lung photographs. (G) Tumor nodule numbers. Results are expressed as individual points (circles) and bars indicate the mean (*n* = 5). *P <* *0.05.

To investigate the effect of decreased ASC expression on malignancy, we adopted the B16BL6 murine melanoma cell line and ablated ASC expression by transduction of the shASC expression gene with retroviral reconstitution. ASC expression was confirmed to be reduced to under one‐fifth of control mRNA and protein levels after hygromycin‐B selection (Fig. 1A and B). However, cell proliferation was not remarkably different from that of controls until 4 days under standard culture conditions (Fig. 1C).

In order to examine tumor formation, B16BL6 cells were inoculated subcutaneously into syngeneic C57BL/6j mice. Tumor growth was comparable in volume and weight between ASC‐knockdown and control cells until the 14‐day endpoint (Fig. 1D and E). Thus, ASC silencing did not visibly affect cellular proliferation either in vitro or in vivo.

To assess the metastatic ability of ASC‐knockdown cells in vivo, B16BL6 cells were injected into the tail vein of syngeneic mice, and the number of metastatic nodules formed in lungs was counted 2 weeks afterward. As there were significantly more metastatic foci in mice inoculated with ASC‐knockdown cells than in controls (Fig. 1F and G), diminished ASC expression appeared to increase the metastatic ability to B16BL6 cells in this in vivo model system.

### ASC silencing significantly enhanced the motility of B16BL6 cells

**Figure 2:**
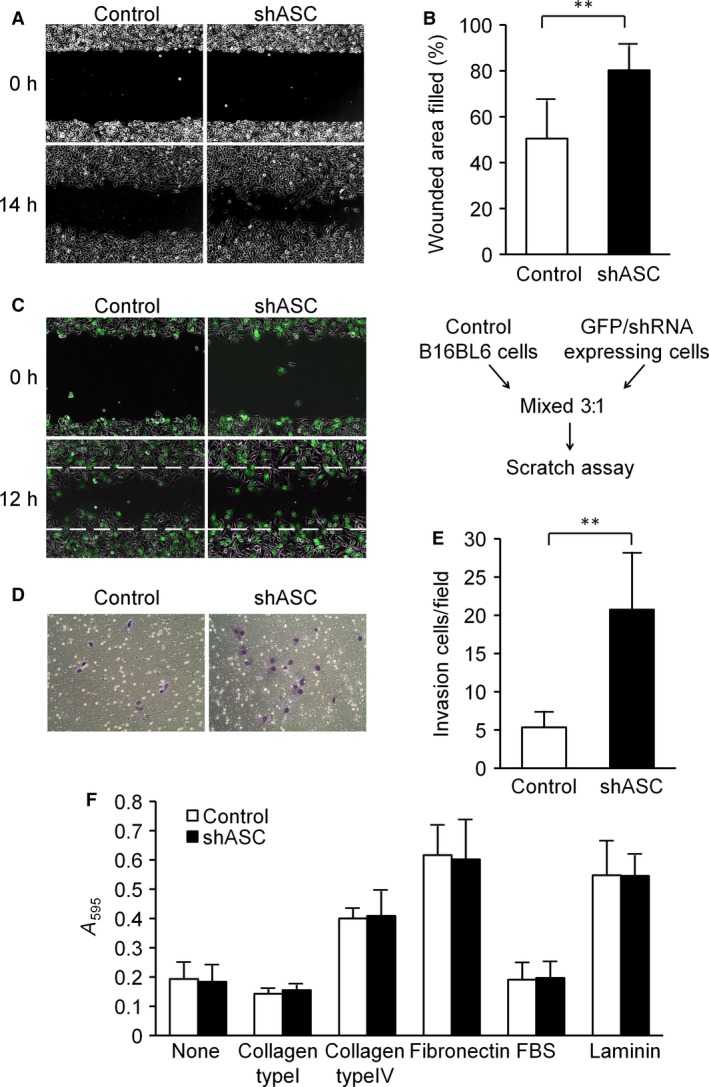
ASC silencing significantly enhanced the motility of B16BL6 cells. (A and B) Scratch assay. (A) Representative photographs taken immediately (0 h) and 14 h after wounding. (B) The wounded areas were measured by ImageJ software, and the percent of wound closure was calculated for each area. Results are expressed as the mean (*n* = 6), and bars indicate SD. **P <* *0.01. (C) Scratch assay with GFP‐labeled cells. Parental and GFP/shRNA‐expressing cells were mixed at a ratio of 3: 1 and then seeded in the same manner as in experiment A. Migration progress was photographed immediately (0 h) and 12 h after wounding under a fluorescent‐inverted microscope. Representative results are shown. (D and E) Transwell invasion assay. (D) Representative photographs of invading cells. (E) Invading cells were counted in five randomly chosen fields in each wells. Results are expressed as the mean of triplicate counts (*n* = 15) and bars indicate SD. **P <* *0.01. F. ECM adhesion assay. After pre‐coating 96‐well plates with the indicated ECM molecules, adhesion assays were performed as described in Materials and Methods. Results are expressed as the mean (*n* = 4) and bars indicate SD.

Since Liu et al. 16 reported that ASC overexpression suppressed cell migration and invasion in RCC cell lines, we next examined the effects of ASC‐knockdown on the cellular motility of B16BL6 cells in scratch assays. Interestingly, cellular motility, or wound healing, was significantly enhanced in ASC‐knockdown cells within 14 h of scratching (Fig. 2A and B). We further evaluated this enhancement in cell migration by ASC ablation by mixing GFP‐expressing ASC‐knockdown cells with control cells (Fig. 2C). The GFP‐labeled cells migrated visibly more than controls (Fig. 2C), which suggested that upregulation of cellular motility by ASC‐knockdown was caused by intracellular alterations and not by secreted molecules. Moreover, no differences were noted additional analyses of the mRNA expression levels of cytokines or chemokines related to cancer progression and metastasis (Fig. S1). We also examined the invasion ability of ASC‐knockdown cells by Matrigel invasion assays, which showed that ASC ablation increased the invasiveness of B16BL6 cells (Fig. 2D and E). This result was consistent with the higher migration witnessed in scratch assays.

We next assessed the adhesion of B16BL6 cells to the extracellular matrix (ECM). As shown in Figure 2F, there were no significant differences in adherence onto collagen type I, collagen type IV, fibronectin, FBS, or laminin between ASC‐knockdown cells and controls. As for the *α*5, *α*3, and *β*1 integrins necessary to adhere B16BL6 cells to the ECM, the mRNA expression levels of *α*5 and *α*3 were slightly decreased in ASC‐knockdown cells, and *β*1 integrin remained largely unchanged (Fig. S2). These results were in agreement with a reported loss of *α*3 integrin enhanced keratinocyte migration by Margadant et al. 20.

In cancer cells, cellular motility is often enhanced by epithelial‐mesenchymal transition (EMT) 21. Therefore, we examined the mRNA expression levels of genes related to EMT. After hygromycin‐B selection, Snail mRNA was increased about threefold in ASC‐knockdown cells versus control cells (Fig. S3), although other EMT markers were at comparable levels between cell types (Fig. S3). Moreover, Snail protein expression was not detected (data not shown), and the difference in Snail mRNA expression faded after several passages (data not shown). Taken together, these results suggested that EMT was not related to the motility enhancement of B16BL6 cells in the present system.

### Invadopodia formation was augmented in ASC‐ablated B16BL6 cells

**Figure 3:**
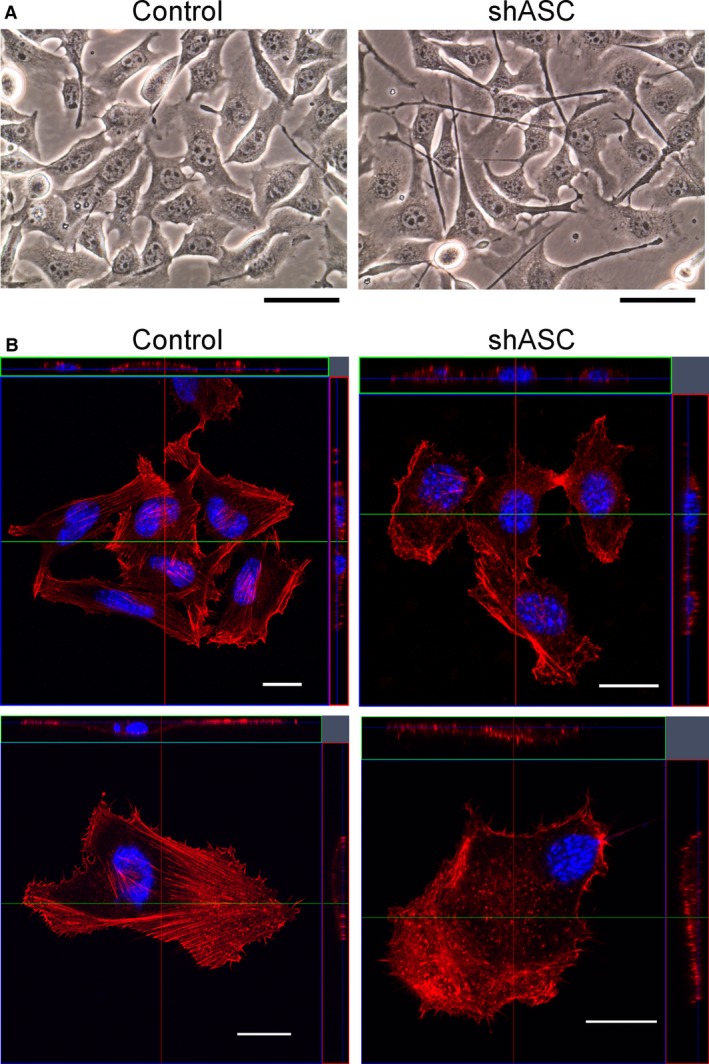
Morphological changes of ASC‐knockdown B16BL6 cells. (A) Phase contrast images of cultivated cells. Original magnification ×400. Scale bars indicate 50 *μ*m. (B) Cells were cultured on fibronectin‐coated coverslips and fixed with 4% PFA. F‐actin was visualized by staining with rhodamine‐phalloidin and observed under a confocal microscope. Representative photographs are shown. Scale bars indicate 20 *μ*m.

Microscopically filopodia‐like dendritic protrusions were detected under standard culture conditions as a morphological change in ASC‐knockdown cells (Fig. 3A). As cellular motility has been enhanced by ASC ablation (Fig. 2), we expected cellular cytoskeletal changes to accompany these phenotypic alterations. F‐actin staining with rhodamine‐conjugated phalloidin showed that actin stress fibers were relatively well organized in control cells as compared with ASC‐knockdown cells, wherein fibers were seen in lamellipodia‐like and dot‐like structures (Fig. 3B). Furthermore, dot‐like clusters of F‐actin indicative of invadopodia formation were enhanced in ASC‐knockdown B16BL6 cells.

**Figure 4:**
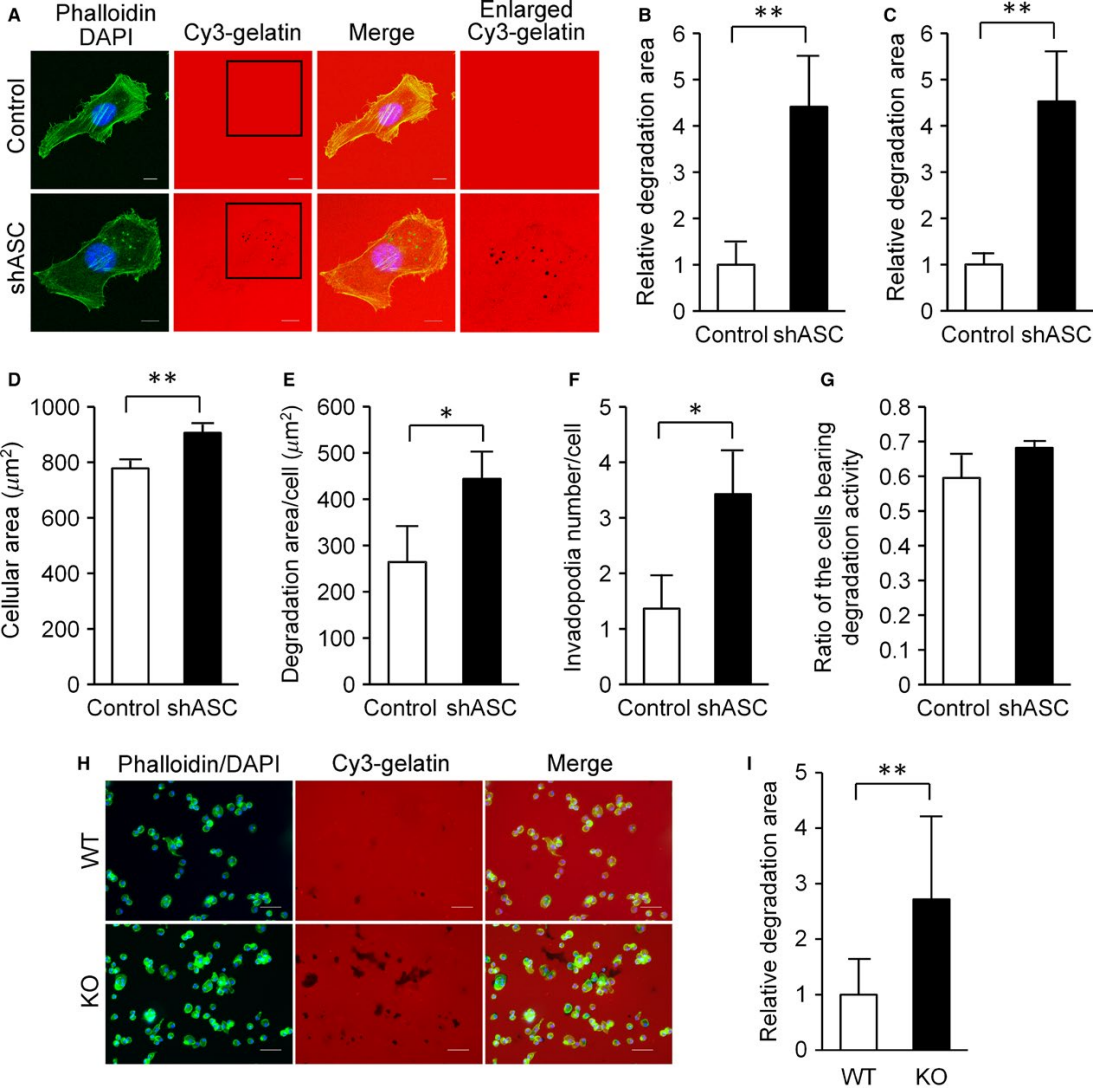
Elevated invadopodia formation in ASC‐ablated cells. (A) Cells were cultivated on Cy3‐gelatin‐coated Lab‐Tec chamber slides for 5 h and fixed with 4% PFA in PBS prior to staining with FITC‐phalloidin and DAPI. Fluorescent signals were observed under a confocal microscope. Representative results are shown. Enlarged areas of squares in Cy3‐gelatin photographs are presented. Scale bars indicate 10 *μ*m. (B) To quantitate the gelatin degradation activity of invadopodia, signals were observed under a fluorescence microscope, analyzed with ImageJ software, and normalized to total cell number in each image. Twenty randomly selected fields were imaged and analyzed for each sample. Results were calculated relative to those of controls. Findings are expressed as the mean (*n* = 20) and error bars indicate SD. (C) The invadopodia assay was performed with B16F10 cells using the same method as in experiment B. (D, E, F, and G) High‐content imaging analysis of invadopodia formation in B16F10 cells. (D) Area of cells. (E) Gelatin degradation area per cells. (F) Number of invadopodia per cell. (G) Ratio of cells bearing gelatin degradation activity. Results are expressed as the mean (*n* = 3) and error bars indicate SD. (H and I) Podosomes of thioglycollate‐induced mouse peritoneal macrophages. (H) Representative images of the gelatin degradation activities by mouse peritoneal macrophages. Scale bars, 50 *μ*m. (I) Gelatin degradation areas were determined by the same method as in experiment B. Results were calculated relative to those of WT mice. Results are expressed as the mean (*n* = 20), and error bars indicate SD. *P <* *0.05 and **P <* *0.01.

Originally named after v‐Src‐transformed chicken embryonic fibroblasts 22, invadopodia are known as actin‐based dynamic protrusions 23. We therefore evaluated the formation of invadopodia by gelatin degradation assays as shown in Figure 4A. The dot‐like signals of F‐actin in B16BL6 cells corresponded to sites of gelatin matrix degradation, a distinctive feature of invadopodia (Fig. 4A). Gelatin degradation was clearly more detectable in ASC‐knockdown cells as compared with controls (Fig. 4A), and the degradation area was significantly larger (Fig. 4B). An enhancement in invadopodia formation was also evident when we performed ASC‐knockdown of B16F10 melanoma cells (Fig. 4C).

We further assessed the effects of ASC ablation on invadopodia formation in B16F10 cells with a high‐content imaging system. As shown in Figure 4D, the cellular area of ASC‐knockdown cells was larger than that of controls and consistent with the B16BL6 cell findings (Fig. 3). Figure 4E presents the quantitated data of gelatin degradation seen in Figure 4C by a high‐content imaging system. Furthermore, the number of invadopodia per cell was significantly greater in ASC‐knockdown B16F10 cells than in controls (Fig. 4F). We observed no difference in the number of cells displaying gelatin degradation activity (Fig. 4G). Thus, the enhancement of invadopodia formation appeared to be associated with diminished ASC expression in both B16BL6 and B16F10 cells.

While ECM degradation protrusions are named as invadopodia in cancer cells, they are referred to as podosomes in monocytic cells, endothelial cells, and smooth‐muscle cells 24, 25, 26. We examined the effect of an ASC deficiency on the formation of podosomes using thioglycollate‐induced mouse peritoneal macrophages. As expected, the gelatin degradation area was larger for ASC‐knockout mouse macrophages than for wild‐type macrophages (Fig. 4H and I). Taken together, ASC ablation caused the formation of invadosomes 27, such as invadopodia and podosomes, in cancer cells and macrophages.

### Src and Erk phosphorylations were enhanced in ASC‐knockdown B16BL6 cells

**Figure 5:**
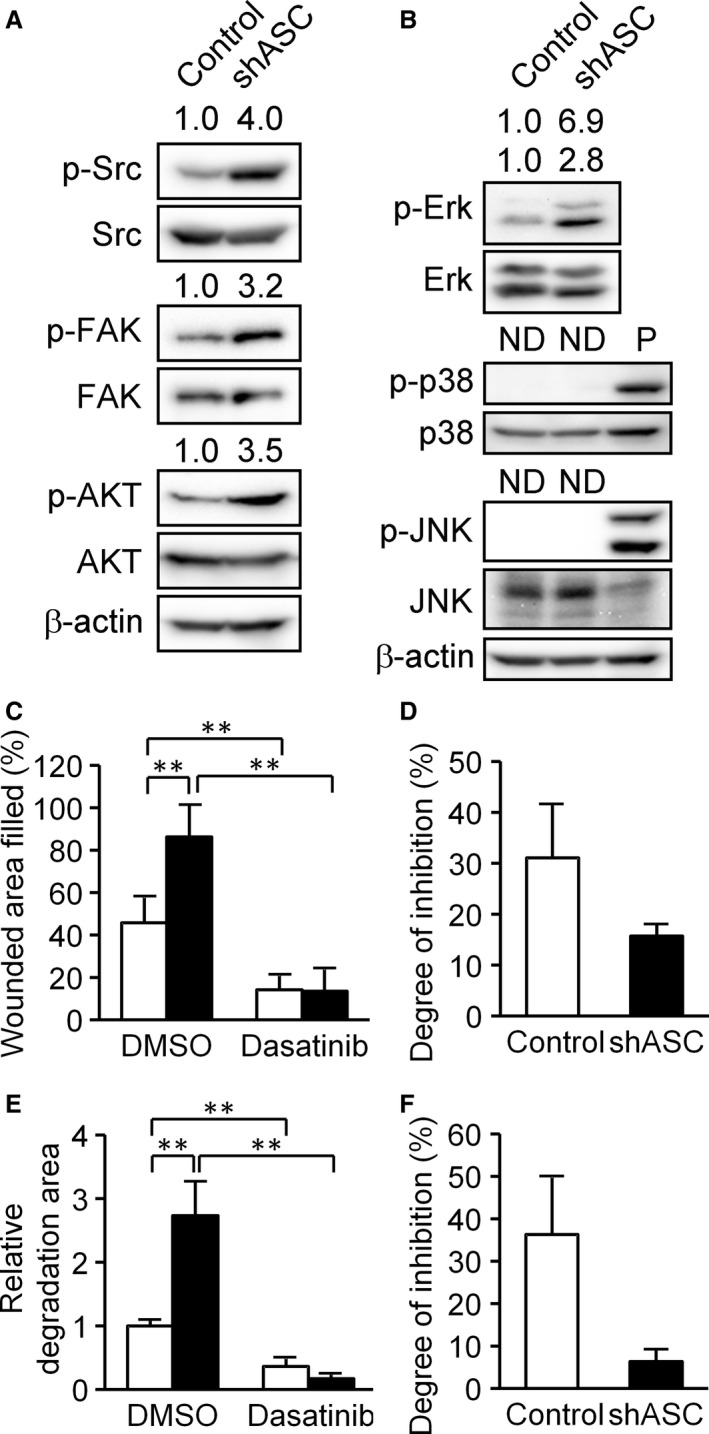
Analysis of intracellular signaling molecules in B16BL6 cells. (A) Phosphorylation patterns of Src (Tyr416), FAK (Tyr397), and Akt (Ser473) in control and ASC knockdown cells. (B) Phosphorylation patterns of Erk 1/2 (Tyr202/Tyr204), p38 MAPK (Tyr180/Tyr182), and JNK (Tyr183/Tyr185). P, positive control; whole‐cell lysates of B16BL6 cells which was treated with 200 ng/mL anisomycin for 30 min. Phosphorylation ratio was calculated by dividing phospho‐protein density with total protein density for each sample and normalized by a control cell value set as 1. Values are indicated on the phospho‐protein samples. ND, not detected. (C) Scratch assays performed with or without 10 *μ*mol/L dasatinib, a potent Src inhibitor. White bars indicate control cells and black bars represent shASC cells. Results are expressed as the mean (*n* = 6), and bars indicate SD. (D) The results of experiment C normalized to the degree of inhibition by dasatinib by dividing the wounded area filled in the presence of dasatinib by that in the absence of dasatinib. (E) Invadopodia assays were performed using the same method as in Figure 4B with or without 10 *μ*mol/L dasatinib. White bars indicate control cells and black bars represent shASC cells. Results are expressed as the mean (*n* = 20) and bars indicate SD. (F) The results of experiment E normalized to the degree of inhibition of gelatin degradation by dasatinib using the same calculation as in experiment D. **P <* *0.01.

We next sought to elucidate the molecular basis of the above phenotypic alterations. Since humoral factors were probably not involved in the ASC‐mediated upregulation of cellular motility, we turned our attention to the intracellular signaling pathways in B16BL6 cells. We initially analyzed the Src signaling pathway as Src is a key molecule in cancer cell migration, invasion 28, and invadopodia formation 22. Western blotting analysis showed that the phosphorylation of Src (Tyr417) was elevated by ASC‐knockdown (Fig. 5A). Moreover, phosphorylation levels of focal adhesion kinase (Tyr397) and Akt (Ser473), both Src signaling pathway molecules, were increased in ASC‐knockdown cells as compared with controls (Fig. 5A). These results suggested that Src signaling pathway enhancement was one of the causes of higher motility and invadopodia formation in ASC‐silenced cells.

We subsequently addressed the phosphorylation of Erk/MAPK, another downstream molecule in the Src signaling pathway 28. As shown in Figure 5B, phosphorylated Erk 1/2 (Tyr202/Tyr204) was apparently enhanced in ASC‐knockdown cells. Erk activation has been reported as essential for melanoma cell invasion and invadopodia formation 29, which was consistent with our results. On the other hand, phosphorylation of p38 MAPK and JNK was not detected (Fig. 5B).

We reperformed the scratch and invadopodia assays with or without dasatinib, a potent Src inhibitor. Dasatinib inhibited the cellular migration of ASC‐knockdown B16BL6 cells to a greater extent than in control cells (Fig. 5C). Specifically, ASC‐knockdown cell migration was suppressed to 15% by dasatinib, versus to ~30% in controls (Fig. 5D). Similar findings were obtained for gelatin degradation ability (Fig. 5E and F), suggesting that ASC‐knockdown cells were more dependent on the Src signaling pathway than control cells. Our cumulative results indicated that ASC ablation enhanced cellular motility and invadopodia formation in B16BL6 cells via modulation of the Src signaling pathway.

### Caspase inhibitors attenuated the cellular motility of ASC‐knockdown cells

**Figure 6:**
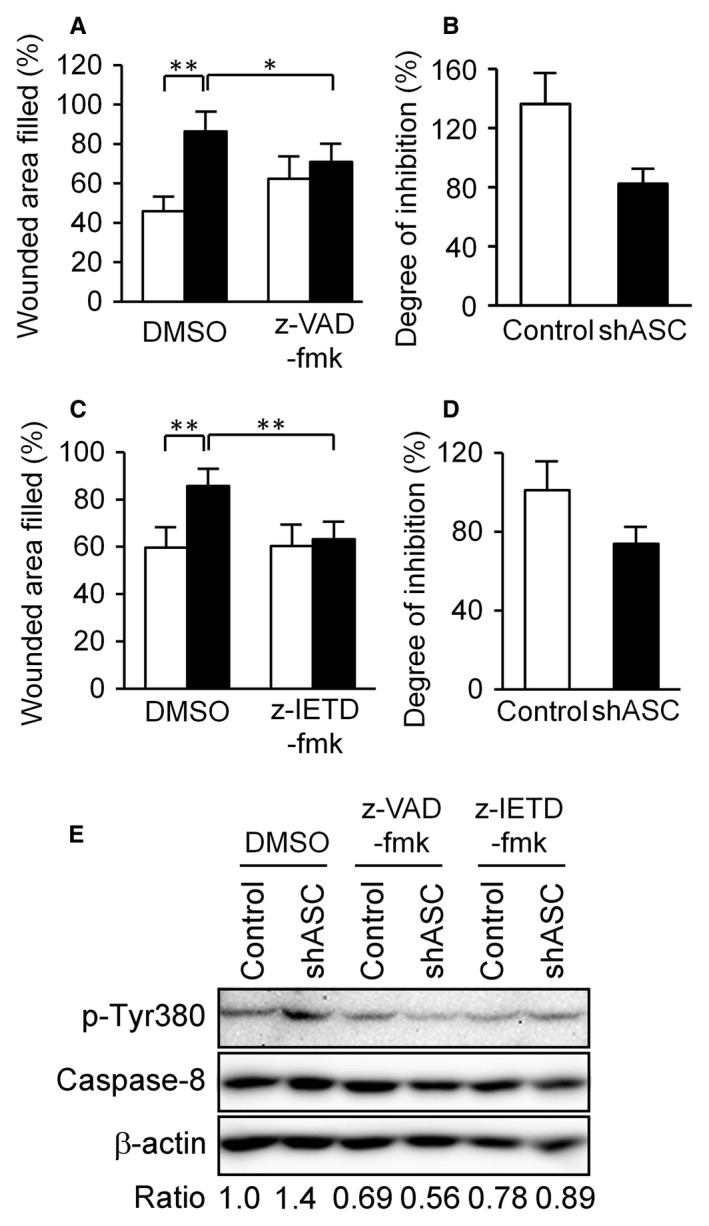
Analysis of the contribution of caspase‐8 to cellular motility. (A) Scratch assays were performed with or without 20 *μ*mol/L z‐VAD‐fmk, a pan‐caspase inhibitor. White bars indicate control cells and black bars represent shASC cells. These trials were performed simultaneously with the experiment in Figure 5C. Results are expressed as the mean (*n* = 6) and bars indicate SD. (B) Results of experiment Figure 6A normalized to the degree of inhibition by z‐VAD‐fmk by dividing the wounded area filled in the presence of z‐VAD‐fmk by that in the absence of z‐VAD‐fmk. (C and D) Scratch assays were performed with or without 20 *μ*mol/L z‐IETD‐fmk, a caspase‐8‐specific inhibitor. Results are expressed as in A and B. *P <* *0.05 and **P <* *0.01. (E) Western blotting analysis of Tyr380 phosphorylation of caspase‐8. Cells were treated with 20 *μ*mol/L z‐VAD‐fmk or z‐IETD‐fmk for 2 h, attached to 2 *μ*g/mL fibronectin‐coated plates for 30 min, lysed, and evaluated for phosphorylation levels of Tyr380 in caspase‐8 by immunoblotting. Phosphorylation ratio was calculated by dividing phospho‐Tyr380 density with total caspase‐8 density and normalized to a DMSO‐treated control cell value set as 1.

Recent reports have described that caspase‐8, a key mediator of apoptosis, also acts as a promoter of cancer cell migration 30, 31 that is converted from an apoptosis initiator to an enhancer of cellular migration by phosphorylation via Src 30, 31. Barbero et al. 32 uncovered that phosphorylation of Tyr380, which is located in the catalytic domain of caspase‐8, was critical for the promotion of cancer cell migration. Accordingly, we examined the effect of a caspase inhibitor, z‐VAD‐fmk, on cell migration and invadopodia formation since it has also been shown that ASC could interact with caspase‐8 33, 34. We observed that z‐VAD‐fmk inhibited the cellular motility enhanced by ASC ablation and slightly affected basal cellular migration ability (Fig. 6A and B). This phenomenon was also observed with the caspase‐8‐specific inhibitor, z‐IETD‐fmk (Fig. 6C and D). In our assessment of Tyr380 phosphorylation of caspase‐8 in ASC‐knockdown cells, phosphorylation was enhanced in the knockdown cells but attenuated by either z‐VAD‐fmk or z‐IETD‐fmk (Fig. 6E). As z‐VAD‐fmk and z‐IETD‐fmk were reported to bind to the catalytic site of pan‐caspases and caspase‐8, respectively, they may have made Src inaccessible to the caspase‐8 catalytic domain, thereby inhibiting the phosphorylation of Tyr380 and the cellular migration of ASC‐knockdown cells. In invadopodia assays with z‐VAD‐fmk, cells did not attach to gelatin in the presence of the inhibitor and no invadopodia were formed (data not shown). These results suggested that ASC ablation could increase Src accessibility to caspase‐8 for ensuing phosphorylation and cellular migration.

## Discussion

This investigation demonstrated that ASC ablation enhanced cellular motility and invadopodia formation through cytoskeletal reorganization and activation of the Src signaling pathway in the B16BL6 murine melanoma cell line, thus augmenting the metastatic ability of ASC‐silenced cells. The biological ASC function revealed in this study was one of metastatic malignant phenotype suppression, and not altered proliferation ability, which was in agreement with other reports implicating ASC as a tumor suppressor. Incidentally, the ectopic expression of ASC in human fibrosarcoma HT‐1080 cells in which ASC expression is silenced by epigenetic regulation also resulted in the attenuation of cellular motility in scratch assays (our unpublished data). Liu et al. 16 reported that ectopically expressed ASC suppressed RCC cell migration and invasion in 786‐0 and A498 cells. Moreover, we demonstrated a higher experimental metastatic ability of ASC‐knockdown B16BL6 cells to the lung. Based on these lines of evidence, the suppression of ASC in human cancers may be a key factor contributing to a poor prognosis.

Since ASC ablation was seen to increase phosphorylation levels in Src family kinases (Fig. 5), we first assessed Src signaling activation by evaluating Src family kinases by immunoprecipitation analysis. However, no associations were detected between ASC and the Src family kinases of c‐Src, Hck, Yes, Fyn, or Lyn (data not shown). We next examined the signaling molecules related to Src phosphorylation. It is known that Src family kinases are regulated by receptor‐type tyrosine kinases (RTKs) 35 and MET tyrosine kinase, also known as HGF receptor 36, 37. No alterations in MET or HGF expression were seen in ASC‐knockdown B16BL6 cells (Fig. S4). Other modulation candidates of Src phosphorylation were protein phosphatase PTP1B 38 and SHP‐2 39, whose mRNA levels were also remained unchanged by ASC ablation (Fig. S5). Elsewhere, intracellular reactive oxygen species (ROS) were reported to activate Src tyrosine kinase 40, so we evaluated intracellular ROS in ASC‐knockdown cells by CM‐H_2_DCFDA staining. ROS level did not differ significantly between ASC‐knockdown and control cells (Fig. S6). Thus, we have yet to pinpoint the cause of elevated Src phosphorylation in ASC‐knockdown cells. Proteomic analysis is currently underway to identify ASC‐binding proteins in cancer cells, but the molecules described above, such as MET and PTP1B, have not provided any clues. Further analysis is necessary to clarify the mechanisms of Src phosphorylation by ASC silencing.

When cytoskeletal remodeling‐related molecules were surveyed, we observed that ASC colocalized with the cytoskeletal modulating protein IQGAP1 in B16BL6 cells in immunofluorescence assays (Fig. S7). It has been reported that IQGAP1 binds to the GTP‐bound form of Cdc42 and Rac1 with substantially high affinity and localizes in invadopodia as a key molecule of matrix degradation 41. Although we searched for interactions between ASC and IQGAP1 in B16BL6 cells by immunoprecipitation, none were detected (data not shown), suggesting ASC to be weakly associated or indirectly complexed with IQGAP1. Since IQGAP1 has also been linked to RAF, MEK1/2, and Erk1/2 cascade kinases as a MAPK scaffolding protein 42, the higher Erk1/2 phosphorylation seen in ASC‐knockdown cells may also have reflected a weak or indirect interaction of ASC with IQGAP1. Waite et al. 43 reported that in COS‐7 cells, ASC and actin co‐localized at cellular sites rich in polymerizing actin. They also suggested that Pyrin recruited the cytoskeletal‐related molecules PSTPIP1, Arp3, and VASP to ASC specks 43. If these molecules, including IQGAP1 and other cytoskeletal components, interact with ASC in cancer cells, ASC may have a tumor suppressive role as a regulator of cytoskeletal remodeling.

According to Liu et al., 44 ASC has dual, stage‐dependent roles in tumorigenesis by regulating NF‐*κ*B activity and IL‐1*β* secretion in human melanoma cells. Here, Western blotting analysis showed that while NF*κ*B p65 expression level was higher in ASC‐knockdown cells than in controls, its phosphorylation was not detected in either cell (Fig. S8A). I*κ*B*α* protein level measurements in these cells also did not reveal any notable differences (Fig. S8A), and NF*κ*B activity analysis by luciferase reporter assays showed no significant differences between ASC‐knockdown and control cells (Fig. S8B). As it has been reported that TNF‐*α* administration enhanced the incidence of metastasis in several animal models 45, 46, we stimulated B16BL6 cells with TNF‐*α* and witnessed that ASC‐knockdown cells were more sensitive to TNF‐*α* than controls (Fig. S8B), which suggested that ASC suppression had endowed cells the potential to augment NF*κ*B activity by stimulation with TNF‐*α*. This could have played a role in the increased survival and metastatic ability of B16BL6 cells in vivo to lungs. However, the relationship between NF*κ*B activity and ASC in cancer cells may vary from cell to cell and be modulated by the surrounding microenvironment.

NOD‐like receptors, caspase‐1, and other inflammasome components are well known ASC‐binding proteins in innate immune systems 2, 3, 4. However, we did not detect any mRNA expression of proteins containing an ASC‐binding pyrin domain, such as NLRP1, NLRP3, AIM2, or Pyrin in either B16BL6 or B16F10 cells (data not shown). We also examined caspase‐1 expression in B16BL6 cells, but neither mRNA nor protein expression was detectable (data not shown). Hence, we presume that ASC in cancer cells might have target molecules different from innate immune‐related proteins that act as inhibitors of cytoskeletal remodeling systems, consequently inhibiting invadopodia formation, migration, and other metastatic events.

Lastly, our results demonstrated that a pan‐caspase inhibitor, z‐VAD‐fmk, inhibited the cellular migration of ASC‐knockdown cells (Fig. 6). As caspase‐1 expression was not detected in B16BL6 cells, we considered that other caspases acted as enhancers of cellular motility in ASC‐knockdown B16BL6 cells. It is noteworthy that caspase‐8, a key mediator of apoptosis, also functions as a promoter of cancer cell migration by phosphorylation via Src 30, 31. We observed that the caspase‐8‐specific inhibitor z‐IETD‐fmk inhibited the cellular migration of ASC‐knockdown cells (Fig. 6). Moreover, phosphorylation of Tyr380 in caspase‐8 was elevated by ASC ablation (Fig. 6). Finlay et al. 47 indicated that Src was associated with caspase‐8 through death domain (DED) and enhanced Erk signaling. Barbero et al. 32 reported that phosphorylation of Tyr380 in caspase‐8 was critical for the promotion of cancer cell migration, and explained that caspase‐8 associated with focal adhesion complex, including FAK and Calpain 2, and it promoted tumor cell migration and metastasis 48. Furthermore, it has been reported that ASC bound to caspase‐8 DED through its PYD 33, 34. If ASC binds to caspase‐8 in B16BL6 cells, ASC ablation may have made Src more accessible to caspase‐8, thus enhancing its phosphorylation and ensuing cellular migration. The ASC‐binding protein in cancer cells remains elusive. However, such study may shed light on the relationship between reductions in ASC and tumor progression and contribute to the development of therapeutic systems for metastatic cancers.

## Conflict of Interest

None declared.

## Supporting information


**Figure S1.** Relative mRNA expression of cytokines and chemokines related to metastasis.

**Figure S2.** Relative mRNA expression of integrins necessary to adhere B16BL6 cells to the ECM.

**Figure S3.** Relative mRNA expression of the EMT‐related molecules described in the Figures. Twist was not detected.

**Figure S4.** Relative mRNA expression of RTKs.

**Figure S5.** Relative mRNA expression of major Src phosphatases PTP1B (*Ptpn1*) and SHP‐2 (*Ptpn11*).

**Figure S6.** Intracellular ROS levels of ASC‐knockdown and control cells. (A) Histogram of CM‐H_2_DCFDA‐stained cells by flow cytometry analysis. Red line indicates control cells and green line represents shASC‐transfected cells. (B) Mean fluorescence intensity of CM‐H_2_DCFDA (*n* = 3).

**Figure S7.** Immunocytochemistry of ASC and IQGAP1 in B16BL6 cells. Scale bar indicates 20 *μ*mol/L.

**Figure S8.** ASC‐knockdown enhanced NF*κ*B p65 expression level in B16BL6 cells. (A) Western blot analysis of NF*κ*B‐p65 subunit and I*κ*B. P, positive control; whole‐cell lysates of HeLa cells transfected with poly‐I:C. (B) Reporter assay for NF*κ*B transcriptional activity. FLA and RLA activity was measured using a dual‐luciferae assay kit as described Supplemental Materials and Methods. White bars indicate control cells and black bars represent shASC cells. Results are expressed as the mean (*n* = 3) and error bars indicate SD. **P <* *0.01.

**Data S1.** Methods.
