# Supplementary figures and images for: Novel role of ASC as a regulator of metastatic phenotype
Source: Cancer Med. 2016 Jun 28;5(9):2487–500. doi: 10.1002/cam4.800 (PMC5055161; doi:10.1002/cam4.800)

**Figure S1**

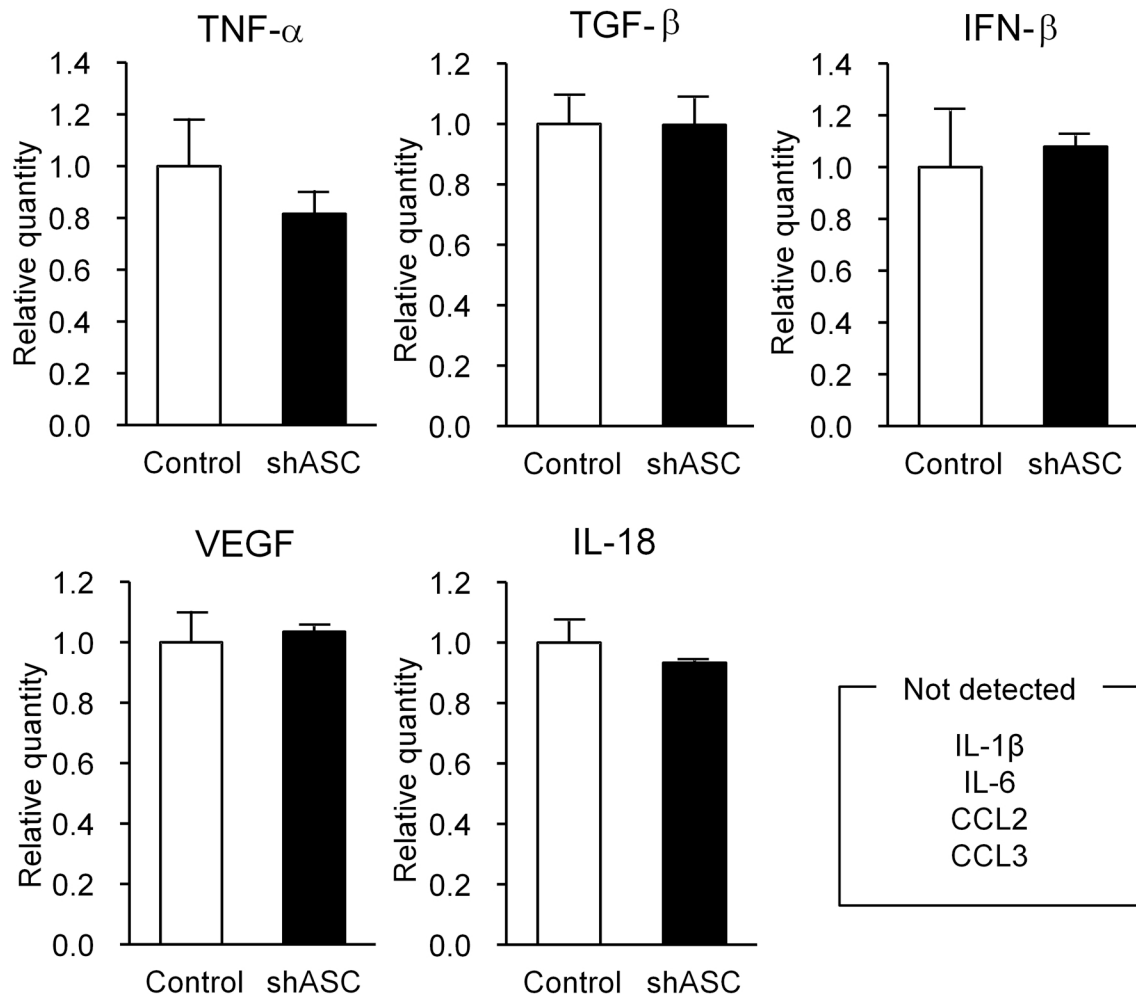

Supplement: Supplementary file 1 — Figure S1. Relative mRNA expression of cytokines and chemokines related to metastasis. [file CAM4-5-2487-s001.pdf]

**Figure S2**

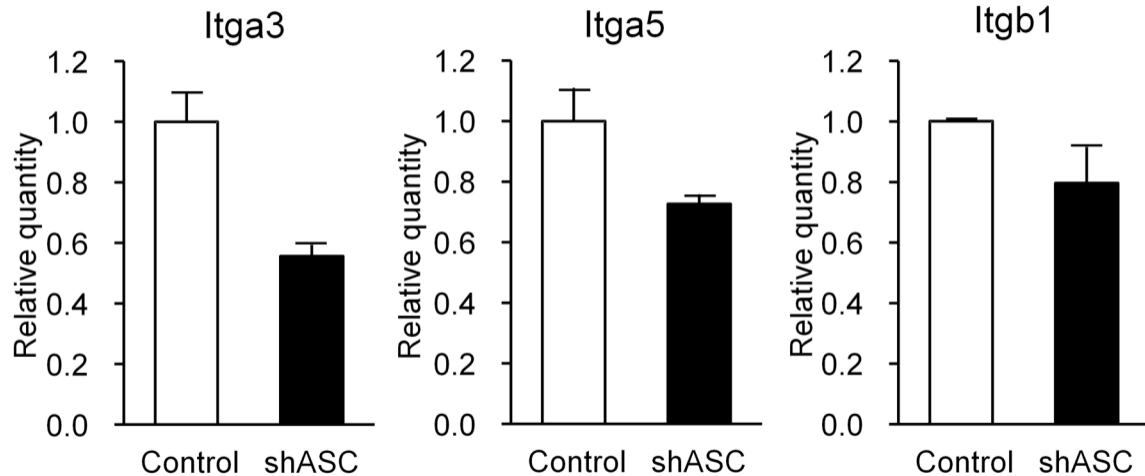

Supplement: Supplementary file 2 — Figure S2. Relative mRNA expression of integrins necessary to adhere B16BL6 cells to the ECM. [file CAM4-5-2487-s002.pdf]

**Figure S3**

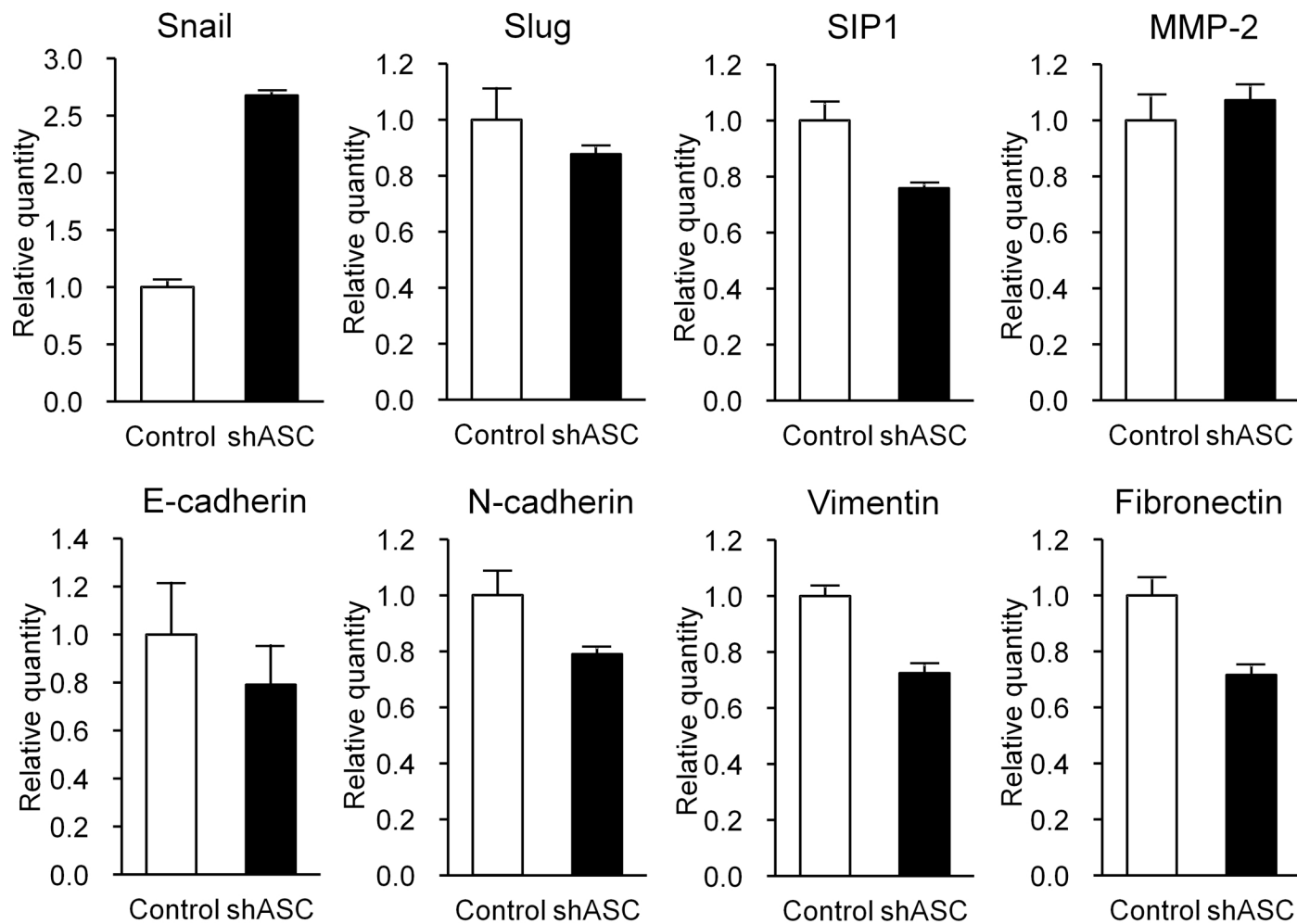

Supplement: Supplementary file 3 — Figure S3. Relative mRNA expression of the EMT‐related molecules described in the Figures. Twist was not detected. [file CAM4-5-2487-s003.pdf]

**Figure S4**

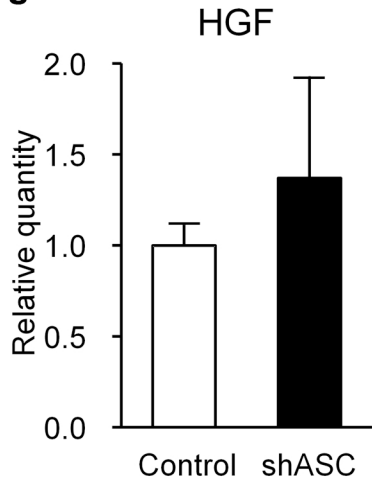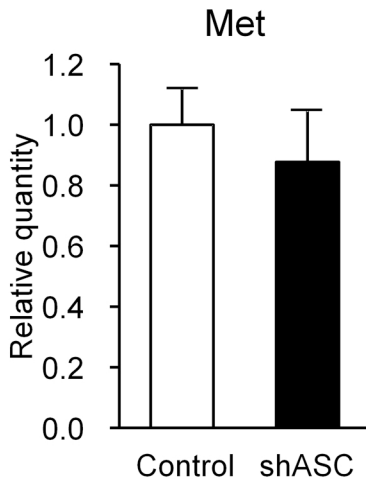

Not detected

EGF  
EGFR

Supplement: Supplementary file 4 — Figure S4. Relative mRNA expression of RTKs. [file CAM4-5-2487-s004.pdf]

**Figure S5**

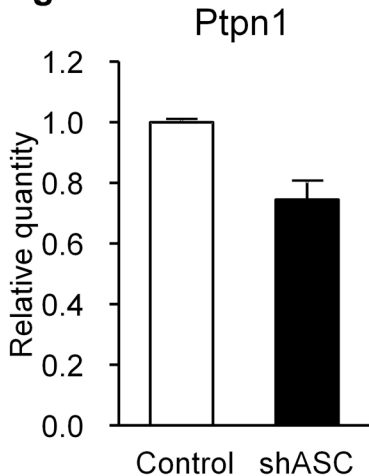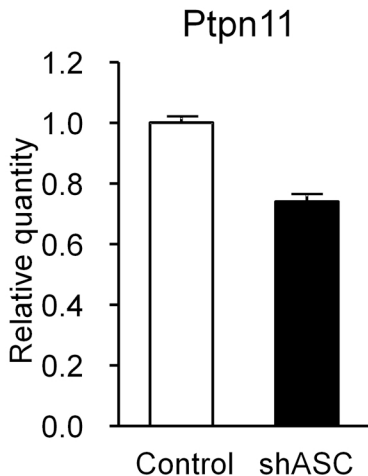

Supplement: Supplementary file 5 — Figure S5. Relative mRNA expression of major Src phosphatases PTP1B (Ptpn1) and SHP‐2 (Ptpn11). [file CAM4-5-2487-s005.pdf]

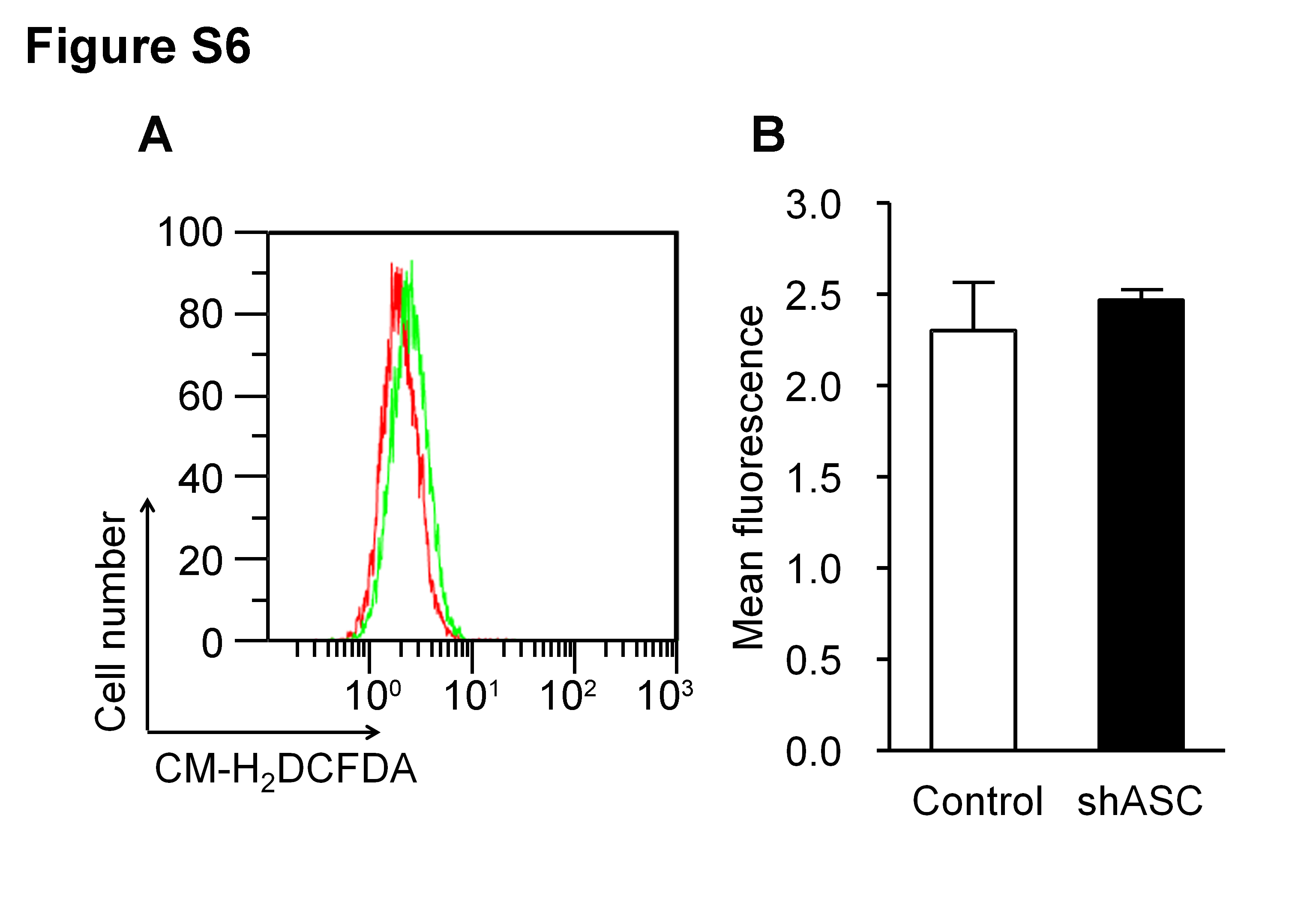

Supplement: Supplementary file 6 — Figure S6. Intracellular ROS levels of ASC‐knockdown and control cells. (A) Histogram of CM‐H2DCFDA‐stained cells by flow cytometry analysis. Red line indicates control cells and green line represents shASC‐transfected cells. (B) Mean fluorescence intensity of CM‐H2DCFDA (n = 3). [file CAM4-5-2487-s006.tif]

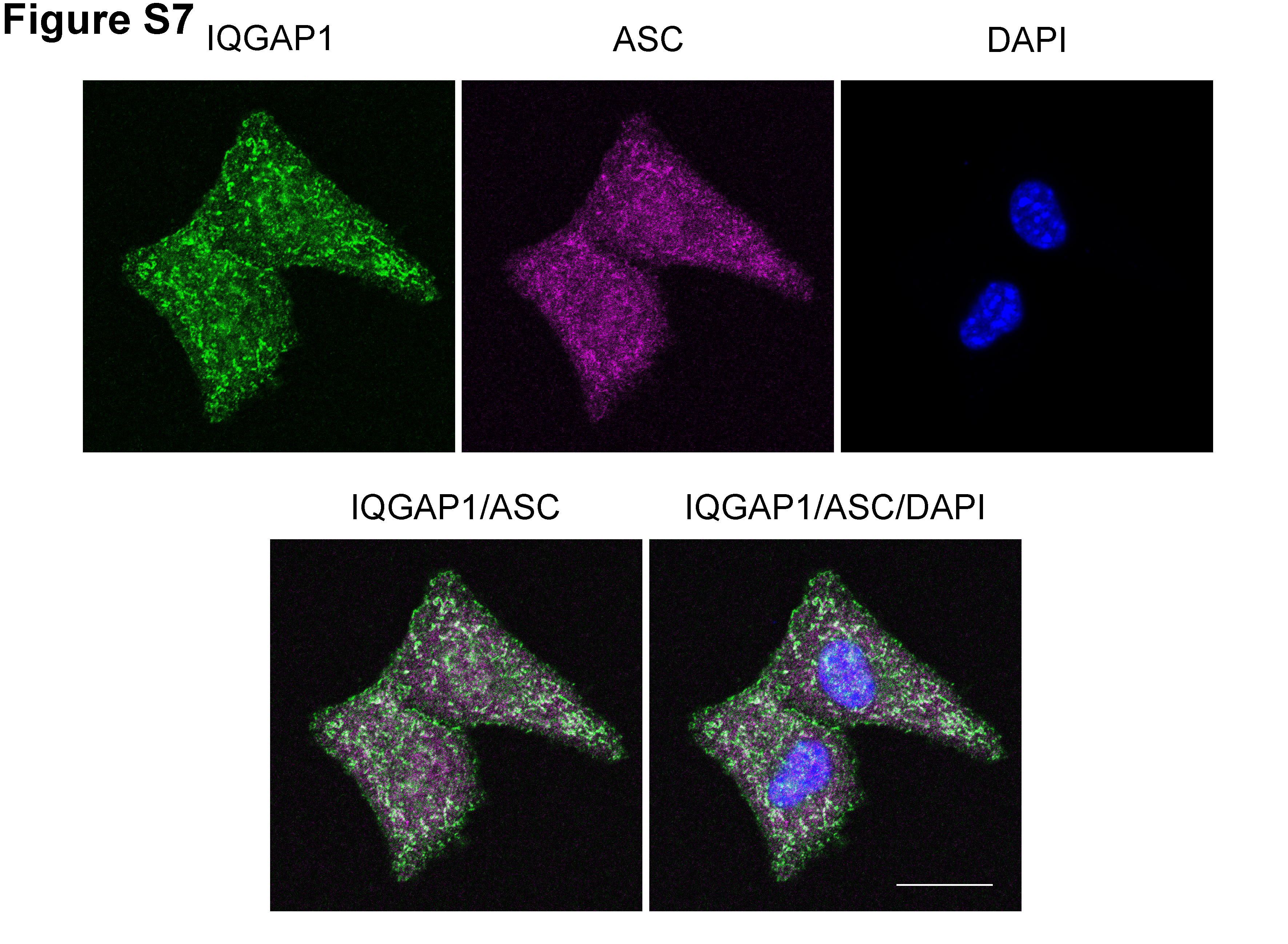

Supplement: Supplementary file 7 — Figure S7. Immunocytochemistry of ASC and IQGAP1 in B16BL6 cells. Scale bar indicates 20 μmol/L. [file CAM4-5-2487-s007.tif]

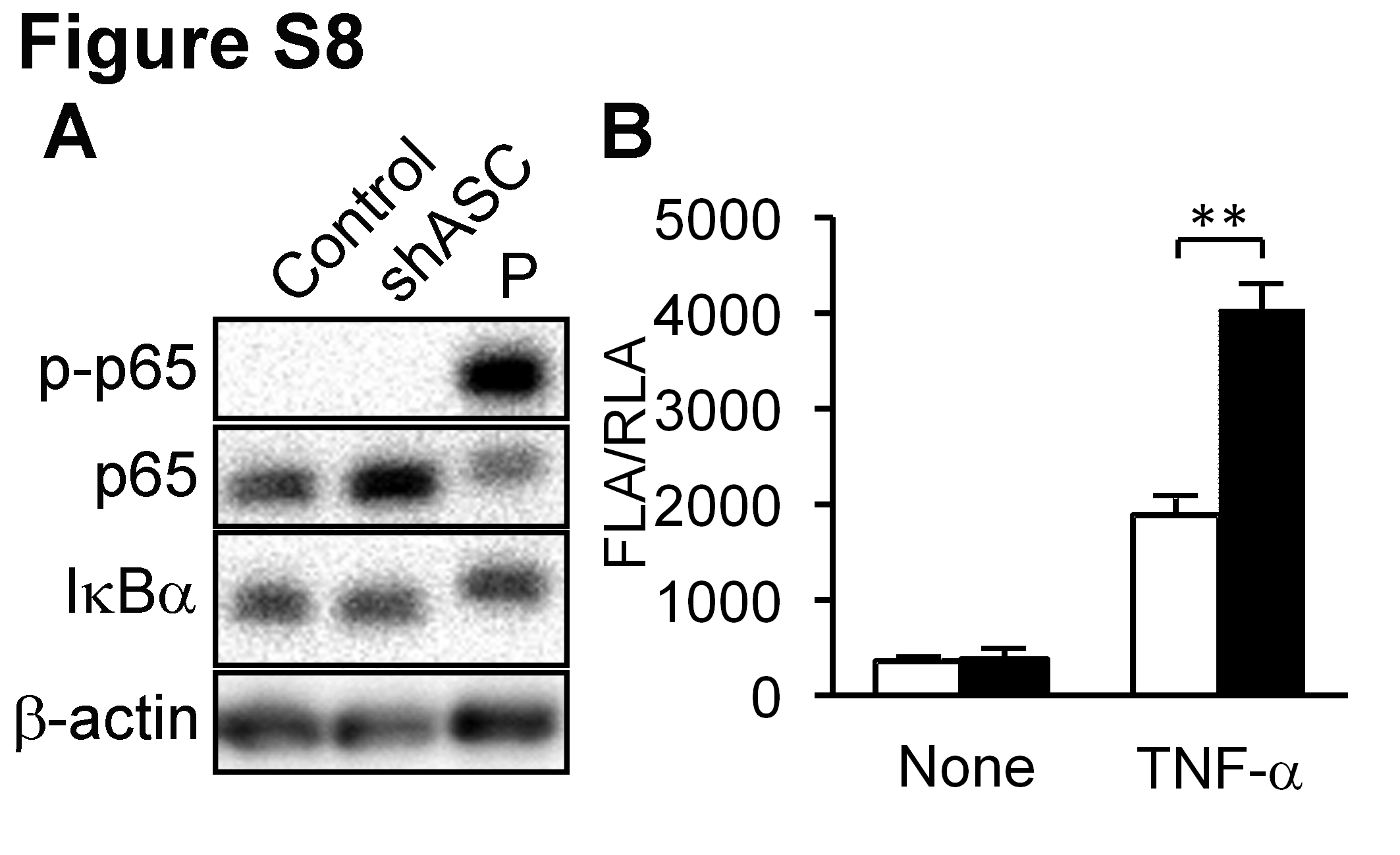

Supplement: Supplementary file 8 — Figure S8. ASC‐knockdown enhanced NFκB p65 expression level in B16BL6 cells. (A) Western blot analysis of NFκB‐p65 subunit and IκB. P, positive control; whole‐cell lysates of HeLa cells transfected with poly‐I:C. (B) Reporter assay for NFκB transcriptional activity. FLA and RLA activity was measured using a dual‐luciferae assay kit as described Supplemental Materials and Methods. White bars indicate control cells and black bars represent shASC cells. Results are expressed as the mean (n = 3) and error bars indicate SD. **P < 0.01. [file CAM4-5-2487-s008.tif]
