# Supplementary material for: Novel role of ASC as a regulator of metastatic phenotype
Source: Cancer Med. 2016 Jun 28;5(9):2487–500. doi: 10.1002/cam4.800 (PMC5055161; doi:10.1002/cam4.800)
Supplement: Supplementary file 9 — Data S1. Methods. [file CAM4-5-2487-s009.doc]

Supporting information

**Supplementary Methods**

*Analysis of ROS*

Quantification of ROS was assessed by flow cytometry using CM-H_2_DCFDA fluorescence dye (Invitrogen, Karlsbad, Germany). Cells were cultivated on 12-well plates for 24 hours, washed with PBS, and incubated with 5 µM CM-H_2_DCFDA in HBSS (+) for 30 min. After CM-H_2_DCFDA incorporation, the cells were incubated in growth medium for 2.5 hours, harvested by trypsinization, washed with PBS, and then analyzed using FACSCanto II (BD Bioscience, Le Pont de Claix, France) and FACSDiva (BD Bioscience) systems along with Kaluza software (Beckman Coulter, Miami, FL).

*Immunocytochemistry*

B16BL6 cells were cultivated on fibronectin-coated coverslips (15 mm in diameter) for 24 hours. The cells were fixed with PBS containing 4% PFA, permeabilized with 0.1% Triton X-100 in PBS, and incubated in in PBS with 1% BSA for 1 hour at room temperature. Samples were treated with anti-IQGAP1 rabbit polyclonal antibodies (Santa Cruz Biotechnology, Santa Cruz, CA) in PBS with 1% BSA over-night at 4 ℃ and thereafter treated with Alexa488-conjugated anti-rabbit IgG in PBS with 1% BSA for 1 hour. The samples were then incubated with Alexa647-conjugated anti-ASC antibodies in PBS with 1% BSA over-night at 4 ℃. Finally, the cells were stained with DAPI, mounted with VECTASHIELD (VECTOR Laboratories), and signals were observed under a con-focal microscope (LSM 5 EXCITER; Carl Zeiss).

*Luciferase assay*

To determine NFκB promoter activity, samples of 4 x 10^4^ cells grown in 24-well plates were transiently transfected with 250 ng of 3 x NFκB promoter fused to the *Firefly Luciferase* reporter gene in pGV-B vectors (Toyo Inki, Tokyo, Japan) and 25 ng of TK-*Renilla* (Promega, Mannheim, Germany) as a control of transfection efficiency. After 48 hours, Luciferase (FLA) and Renilla (RLA) activity was measured using a dual-luciferase assay kit (Promega). For the TNF-α sensitivity analysis, cells were treated with 10 ng/mL recombinant TNF-α (PeproTech EC, London, UK) at 24 hours after transfection.

**Supplementary Figure Legends**

Supplementary Figure S1. Relative mRNA expression of cytokines and chemokines related to metastasis.

Supplementary Figure S2. Relative mRNA expression of integrins necessary to adhere B16BL6 cells to the ECM.

Supplementary Figure S3. Relative mRNA expression of the EMT-related molecules described in the Figures. Twist was not detected.

Supplementary Figure S4. Relative mRNA expression of RTKs.

Supplementary Figure S5. Relative mRNA expression of major Src phosphatases PTP1B (*Ptpn1*) and SHP-2 (*Ptpn11*).

Supplementary Figure S6. Intracellular ROS levels of ASC-knockdown and control cells. A. Histogram of CM-H_2_DCFDA-stained cells by flow cytometry analysis. Red line indicates control cells and green line represents shASC-transfected cells. B. Mean fluorescence intensity of CM-H_2_DCFDA (n = 3).

Supplementary Figure S7. Immunocytochemistry of ASC and IQGAP1 in B16BL6 cells. Scale bar indicates 20 μM.

Supplementary Figure S8. ASC knockdown enhanced NFκB p65 expression level in B16BL6 cells. A. Western blot analysis of NFκB-p65 subunit and IκB. P, positive control; whole cell lysates of HeLa cells transfected with poly-I:C. B. Reporter assay for NFκB transcriptional activity. FLA and RLA activity was measured using a dual-luciferae assay kit as described Supplemental Materials and Methods. White bars indicate control cells and black bars represent shASC cells. Results are expressed as the mean (n = 3) and error bars indicate SD. **, *p* < 0.01.
